# CudaChain: an alternative algorithm for finding 2D convex hulls on the GPU

**DOI:** 10.1186/s40064-016-2284-4

**Published:** 2016-05-21

**Authors:** Gang Mei

**Affiliations:** School of Engineering and Technology, China University of Geosciences, No. 29 Xueyuan Road, Beijing, 100083 China; Institute of Earth and Environmental Science, University of Freiburg, Albertstr. 23B, 79104 Freiburg im Breisgau, Germany

## Abstract

This paper presents an alternative GPU-accelerated convex hull algorithm and a novel *Sorting-basedPreprocessingApproach* (SPA) for planar point sets. The proposed convex hull algorithm termed as CudaChain consists of two stages: (1) two rounds of preprocessing performed on the GPU and (2) the finalization of calculating the expected convex hull on the CPU. Those interior points locating inside a quadrilateral formed by four extreme points are first discarded, and then the remaining points are distributed into several (typically four) sub regions. For each subset of points, they are first sorted in parallel; then the second round of discarding is performed using SPA; and finally a simple chain is formed for the current remaining points. A simple polygon can be easily generated by directly connecting all the chains in sub regions. The expected convex hull of the input points can be finally obtained by calculating the convex hull of the simple polygon. The library *Thrust* is utilized to realize the parallel sorting, reduction, and partitioning for better efficiency and simplicity. Experimental results show that: (1) SPA can very effectively detect and discard the interior points; and (2) CudaChain achieves 5×–6× speedups over the famous Qhull implementation for 20M points.

**Electronic supplementary material:**

The online version of this article (doi:10.1186/s40064-016-2284-4) contains supplementary material, which is available to authorized users.

## Background

The finding of convex hulls is a fundamental issue in computer science, which has been extensively studied for many years. Several classic algorithms have been proposed, including the Graham scan (Graham 1972), the Jarvis’s march (Jarvis 1973), the divide-and-conquer algorithm (Preparata and Hong 1977), the Andrew’s monotone chain (Andrew 1979), the incremental approach (Kallay 1984), and the QuickHull (Barber et al. 1996).

Recently, to speed up the calculating of convex hulls for large sets of points, several efforts have been carried out to redesign and implement several commonly used CPU-based convex hull algorithms on the GPU. For example, Srikanth et al. (2009) and Srungarapu et al. (2011) parallelized the QuickHull algorithm (Barber et al. 1996) to accelerate the finding of two dimensional convex hulls. Based on the QuickHull approach, Stein et al. (2012) presented a novel parallel algorithm for computing the convex hull of a set of points in 3D using the CUDA programming model. Tang et al. (2012) developed a CPU–GPU hybrid algorithm to compute the convex hull of points in three or higher dimensional spaces.

Tzeng and Owens (2012) presented a framework for accelerating the computing of convex hull in the divide-and-conquer fashion by taking advantage of QuickHull. Similarly, White and Wortman (2012) described a pure GPU divide-and-conquer parallel algorithm for computing 3D convex hulls based on the Chan’s minimalist 3D convex hull algorithm (Chan 2003). In Gao et al. (2013), a novel algorithm is proposed to compute the convex hull of a point set in $\hbox {R}^{3}$ by exploiting the relationship between the Voronoi diagram and the convex hull. In addition, Gao et al. (2013) designed ffHull, a flip algorithm that allows nonrestrictive insertion of many vertices before any flipping of edges and maps well to the massively parallel nature of the modern GPU.

When calculating the convex hull of a set of points, an effective strategy for improving computational efficiency is to discard the interior points that have been exactly determined previously. This strategy is referred to as the *preprocessing*/*preconditioning* procedure. The most commonly used preprocessing approach is to form a convex polygon or polyhedron using several determined extreme points first and then discard those points that locate inside the convex polygon or polyhedron; see Stein et al. (2012), Tang et al. (2012), Mei and Xu (2015). The simplest case in two dimensions is to form a convex quadrilateral using four extreme points with min or max *x* or *y* coordinates and then check each point to determine whether it locates inside the quadrilateral; see Akl and Toussaint (1978). Recently, several other strategies are also introduced to efficiently discard interior points (Cadenas and Megson 2014; Xing et al. 2014; Gao et al. 2015).

In this paper, the objective is to design and implement an alternative and efficient convex hull algorithm by exploiting the power of GPU. The contributions in this work can be summarized as follows: (1) a novel and effective *Sorting-based Preprocessing Approach* (SPA) for discarding interior points is proposed; (2) an efficient GPU-accelerated algorithm termed as CudaChain for finding the convex hulls of planar point sets is also presented by utilizing the algorithm SPA .

The proposed convex hull algorithm, CudaChain, consists of two stages: (1) two rounds of preprocessing performed on the GPU and (2) the finalization of calculating the expected convex hull on the CPU. Those interior points that locate inside a quadrilateral formed by four extreme points are first discarded; and then the remaining points are distributed into several (typically four) sub regions. For each subset of points, they are first sorted in parallel; then the second round of discarding is performed using SPA; and finally a simple chain is formed for the current remaining points. A simple polygon can be easily generated by directly connecting all the chains in sub regions. The expected convex hull of the input points can be finally obtained by calculating the convex hull of the simple polygon using the Melkman’s algorithm (Melkman 1987).

The algorithm CudaChain is implemented by heavily taking advantage of the library *Thrust* (Bell and Hoberock 2011) for better efficiency and simplicity. Those very efficient data parallel primitives such as parallel sorting, reduction, and partitioning that are provided by Thrust are directly utilized to implement the CudaChain. The use of the library Thrust makes the implementation easy to develop.

The presented convex hull algorithm, CudaChain, is tested against the Qhull library (Qhull 2015) on various datasets of different sizes using two machines. Experimental results show that CudaChain achieves 5×–6× speedups on average over the Qhull implementation for 20M points. It hopes that this algorithm is an alternative choice in practical applications for the trade-off between its simplicity and efficiency performance.

## Methods

### Algorithm design

The proposed GPU-accelerated algorithm CudaChain is designed on the basis of the fast convex hull algorithm introduced by Akl and Toussaint (1978). The procedure of CudaChain roughly consists of three steps: (1) a first round of preprocessing is first carried out by discarding those points locating inside a quadrilateral formed by four extreme points. This commonly used strategy of preprocessing was described in Akl and Toussaint (1978); (2) the remaining points are distributed into several (typically four) sub regions; and those points in the same region are sorted according to their coordinates; then a novel *Sorting-based Preprocessing Approach* (SPA) is performed to further discard interior points for each sub region and form a simple polygon; (3) the Melkman’s algorithm (Melkman 1987) is finally employed to calculate the convex hull of the simple polygon. The obtained convex hull is exactly the expected convex hull of the input point set. The first and second steps of CudaChain are performed on the GPU, while the third is carried out on the CPU.

More specifically, the procedure of the proposed algorithm is listed as follows:

1. Find four extreme points that have the max or min *x* and *y* coordinates by parallel reduction, denote them as $P_{minx}$, $P_{maxx}$, $P_{miny}$, and $P_{maxy}$
2. Determine the distribution of all points in parallel, and discard the points locating inside the quadrilateral formed by $P_{minx}$, $P_{miny}$, $P_{maxx}$, and $P_{maxy}$
3. Denote the subset of points locating in the four sub regions, i.e., the lower left, lower right, upper right, and upper left as $\hbox {S}_{R1}$, $\hbox {S}_{R2}$, $\hbox {S}_{R3}$, and $\hbox {S}_{R4}$, respectively
4. Sort $\hbox {S}_{R1}$, $\hbox {S}_{R2}$, $\hbox {S}_{R3}$, and $\hbox {S}_{R4}$ separately in parallel; see Table 1 for the orders of sorting
5. Perform the SPA for $\hbox {S}_{R1}$, $\hbox {S}_{R2}$, $\hbox {S}_{R3}$, and $\hbox {S}_{R4}$ to discard interior points further, and form a simple chain for the remaining points in each sub region
6. Form a simple polygon by connecting those four chains in counterclockwise (CCW)
7. Find the convex hull of the simple polygon using Melkman’s algorithm (Melkman 1987).

In the above procedure, the most commonly used strategy for discarding interior points is first carried out (i.e., the Steps 1 and 2); and then those remaining points are divided into four subsets. After that, each subset of points is sorted separately. The key step in this procedure is the second round of discarding interior points and the forming of a simple chain for each subset. A simple polygon can be easily created by directly connecting the chains; and the expected convex hull can be found using Melkman’s algorithm (Melkman 1987) which is specifically designed for calculating the convex hull of a simple polygon.

**Fig. 1:**
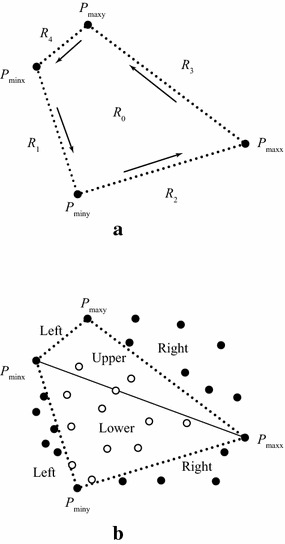
The distribution of points and the first round of discarding. **a** The four extreme points; **b** The four sub-regions

*Step 1: Points’ distribution and the first round of discarding* The strategy of discarding the interior points locating inside a quadrilateral formed by four extreme points is straightforward; see Fig. 1. There is no need to describe this strategy in more details. The only remarkable issue is that: to reduce the computational cost, in the process of checking whether a point is interior (i.e., locating in the region $\hbox {R}_{0}$ in Fig. 1a), the distribution of those non-interior points can also be easily determined. For all points, the following simple method is adopted to determine their distributions:

1. if point *P* lies on the right side of the directed line $P_{minx}P_{miny}$, then it falls in the region $\hbox {R}_{1}$;
2. else if *P* lies on the right side of the directed line $P_{miny}P_{maxx}$, then it falls in the region $\hbox {R}_{2}$;
3. else if *P* lies on the right side of the directed line $P_{maxx}P_{maxy}$, then it falls in the region $\hbox {R}_{3}$;
4. else if *P* lies on the right side of the directed line $P_{maxy}P_{minx}$, then it falls in the region $\hbox {R}_{4}$;
5. else *P* falls in the region $\hbox {R}_{0}$.

After the above procedure of determination, all points are distributed into five regions. Those points in the region $\hbox {R}_{0}$ are interior ones, and need to be directly discarded in this step, while the remaining points in the other four regions should be taken into consideration for calculating the convex hull.

**Fig. 2:**
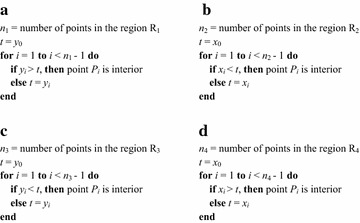
The rules for discarding interior points of the SPA. **a** Rules for discarding points in the low left sub-region; **b** Rules for discarding points in the low right sub-region; **c** Rules for discarding points in the upper right sub-region; **d** Rules for discarding points in the upper left sub-region

*Step 2: Second round of discarding and forming simple polygon* This section will describe a novel sorting-based preprocessing approach that is specifically applicable to the previously sorted points. This method is termed as the SPA. The rules for discarding interior points in those four sub regions, i.e., lower left ($\hbox {R}_{1}$), lower right ($\hbox {R}_{2}$), upper right ($\hbox {R}_{3}$), and upper left ($\hbox {R}_{4}$), are presented in Fig. 2 and Table 1.

**Fig. 3:**
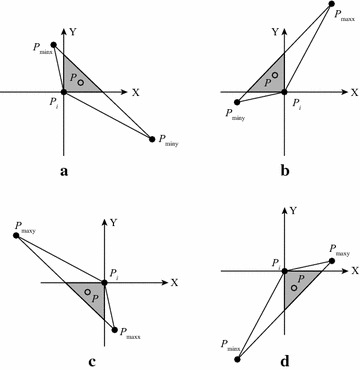
Demonstrations for the correctness of discarding interior points in the method SPA. **a** The region $\hbox {R}_1$; **b** the region $\hbox {R}_2$; **c** the region $\hbox {R}_{3}$; **d** the region $\hbox {R}_{4}$

The correctness of SPA for each sub region is demonstrated in Fig. 3. For the region $\hbox {R}_1$, the first point and the last point are the $P_{minx}$ and $P_{miny}$, respectively; see Fig. 3a. Assuming the point $P_{i}$ has been determined to be non-interior, and now it is checking the point *P* according to the relationship between $P_i$ and *P*. Since all the points in the region $\hbox {R}_{1}$ have been sorted in the ascending order of *x* coordinates, thus $x_P > x_{Pi}$; and if $y_{Pi}$ is larger than $y_P$, then the point *P* must be located in the shaded triangular area; and obviously it also falls in the triangle formed by three points $P_i$, $P_{miny}$, and $P_{minx}$. Hence, the point *P* must be an interior one and needs to be discarded. The correctness of SPA for other three regions can also be explained similarly.

**Fig. 4:**
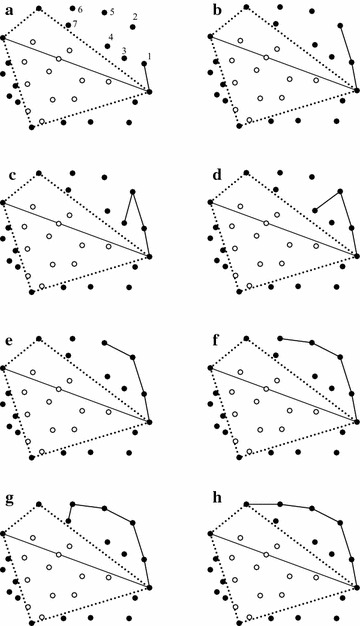
A simple example of forming the chain in the upper left region (The eight steps of forming the chain in the upper left region are illustrated in the subfigures **a**–**h**, respectively)

The forming of the chain in the upper left region ($\hbox {R}_{3}$) is also illustrated as an example; see Fig. 4. Previously, seven points have been sorted in the descending order of *x*. The point $P_{1}$ is first checked; and obviously it is not an interior point according the rule presented in the Fig. 2c. Similarly, it is also found that the point $P_2$ is an exterior point and needs to be kept. However, the point $P_3$ is an interior point since its *y* coordinate is less than that of the point $P_2$; and obviously, the point $P_3$ locates inside the triangle formed by the first point (i.e., the point $P_{maxx}$), the last point (i.e., the point $P_{maxy}$), and $P_2$. After discarding the point $P_3$, the point $P_4$ can also be discarded, while both the points $P_5$ and $P_6$ are not exterior points. However, for that the coordinate *y* of the point $P_7$ is less than that of the point $P_6$, it also should be removed.

**Fig. 5:**
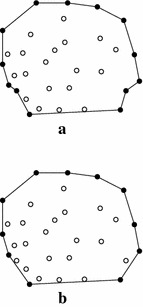
The convex hull of a simple polygon. **a** A simple polygon; **b** The desired convex hull

*Step 3: Calculating the convex hull of simple polygon* the output of the previous step is a simple polygon, which is also an approximate convex hull. To calculate the exact convex hull of the input point set, the fast algorithm introduced by Melkman (1987) is chosen to compute the convex hull of the simple polygon. The convex hull of the simple polygon is the convex hull of the input data set; see Fig. 5.

### Proof of correctness

As described in “Algorithm design” section, the proposed convex hull algorithm is composed of two stages, and has three main steps, including two rounds of preprocessing procedures on the GPU and the finalization of computing the expected convex hull on the CPU. Here the correctness of each step is analyzed to demonstrate the correctness of the entire algorithm.

#### The first round of preprocessing

This pass of preprocessing is carried out to discard those points that locate inside a convex quadrilateral formed by four extreme points with min or max *x* or *y* coordinates. This preconditioning method is first introduced by Akl and Toussaint (1978), and is also used as the initiation/first step in the famous convex hull algorithm, QuickHull (Barber et al. 1996), for two-dimensions. According to the definition of convex hull, the correctness of this preprocessing procedure can be obviously guaranteed since the points locating inside a convex polygon formed by other points are not the extreme points and thus can be directly discarded.

#### The second round of preprocessing (SPA)

This pass of preprocessing is performed to further discard interior points. The basic idea behind the SPA is to identify interior/non-extreme points according to the monotonicity of *x* and *y* coordinates for the remaining points in each sub region. The following two efforts are carried out to demonstrate the correctness of SPA:

1. It will be proved that: in each sub region there exists monotonicity of the *x* and *y* coordinates for the extreme points of a *Convex Hull*.
2. It will be also proved that: if those points in each sub region do not satisfy the property of being both *x*- and *y*-monotone, then they are non-extreme points and thus can be discarded.

The second effort for proving the correctness has been presented in “Algorithm design” section. Here it only proves that: in each sub regions, there exists monotonicity of the *x* and *y* coordinates for the extreme points of a convex hull.

Before proving the correctness of discarding interior points using SPA, the following definitions are given. Note that the definitions of monotone polygon and monotone chain slightly differ from the corresponding versions presented in Chan (1996).

- *Simple polygon* A closed region of the plane enclosed by a simple cycle of straight line segments. A polygon is simple if it contains no holes, i.e., its boundary consists of a single closed chain.
- *Monotone polygon* “A simple polygon is monotone if there exist two extreme vertices in a preferred direction (such as $P_{ymax}$, $P_{ymin}$ if the *y* direction is preferred) such that they are connected by two polygonal chains monotonic in this direction (Toussaint 1984).”
  A chain is monotone with respect to a straight line ${{\varvec{l}}}$ where the vertices in the chain and their projections on ${{\varvec{l}}}$ can appear in the same order. A chain or polygon that is monotone with respect to the *y*-axis is called *y*-monotone.
- *Convex polygon* A simple polygon in which any two boundary points can be joined by a segment that lies completely within *P* .
  The internal angle at each boundary point of a convex polygon is less than 180 degrees. A convex polygon is monotone with respect to all directions (Preparata and Shamos 1985).
- *Convex hull in 2D* The convex hull of a finite set of points *S* in the plane is the smallest convex polygon *P* that encloses *S* .

According to the above definitions, the following relationship can be easily obtained: Convex hull in 2D $\subset$ Convex polygon $\subset$ Monotone polygon $\subset$ Simple polygon, where the symbol $\subset$ means “belongs to” or “is contained in”.

##### **Lemma 1**

*A convex hull in 2D is monotone with respect to all directions.*

##### *Proof*

It has been proved that a convex polygon is monotone with respect to all directions (Preparata and Shamos 1985). Therefore, as one type of convex polygon, a convex hull in 2D is also a monotone polygon with respect to all directions. $\square$

##### **Lemma 2**

*The polygonal chains obtained by dividing a 2D convex hull using the leftmost, bottommost, rightmost, and topmost extreme points are bothx- andy-monotone.*

##### *Proof*

**Fig. 6:**
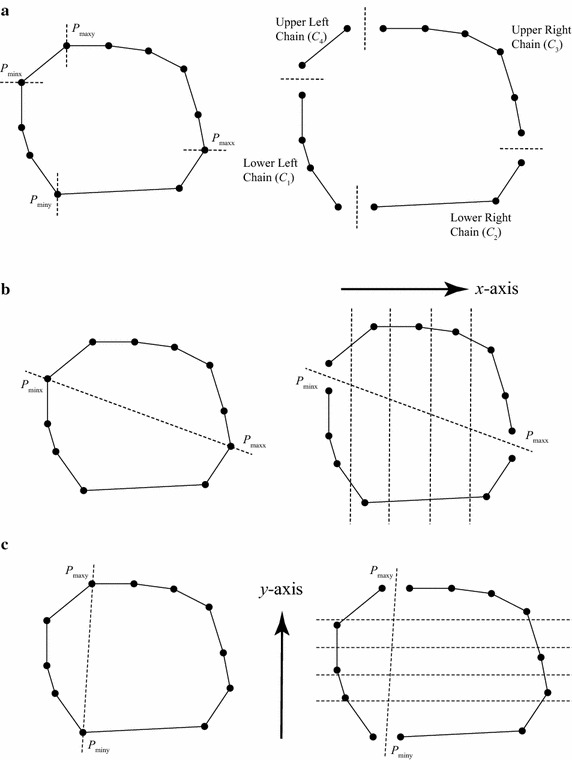
Demonstration of monotonicity. **a** Dividing of chains, **b** Proving of *x*-monotonicity, **c** Proving of *y*-monotonicity

Typically, four polygonal chains can be obtained by dividing a 2D convex hull using the above mentioned four extreme points. Here it only considers this general case for this proving. Let $C_1$, $C_2$, $C_3$, and $C_4$ denote the lower left, lower right, upper right, and upper left chains, respectively; see Fig. 6a.

First, it will prove that the four polygonal chains are *x*-monotone. Recall that a convex hull is monotone with respect to any directions such as *x* and *y*; see Lemma 1. In other words, a convex hull is both *x*-monotone and *y*-monotone. When the *x* direction is preferred, then according to the definition of monotone polygon described above, two extreme points with respect to the preferred *x* direction, i.e., the leftmost point ($P_{minx}$) and rightmost point ($P_{maxx}$), can be used to split the boundary of convex hull into two *x*-monotone chains (i.e., the lower and the upper) such that the *x*-coordinates of the points of a single polygonal chain are monotonically increasing or decreasing; see Fig. 6b. Obviously, the lower left chain ($C_1$) and the lower right chain ($C_2$) are part of the lower monotone polygonal chain, and thus are *x*-monotone. Similarly, because both the upper left chain ($C_3$) and the upper right chain ($C_4$) are derived from the upper monotone polygonal chain, they are *x*-monotone.

Second, it will prove that the four polygonal chains are *y*-monotone. Similar to the first step of proving, by using two extreme points with respect to the preferred *y* direction (i.e., $P_{miny}$ and $P_{maxy}$), a convex hull can be also decomposed into two monotone polygonal chains (i.e., the left and the right) such that the *y*-coordinates of the points of a single polygonal chain are monotonically increasing or decreasing; see Fig. 6c. The lower left ($C_1$) and the upper left ($C_4$) are part of the left monotone polygonal chain, and thus are *y*-monotone, while the lower right chain ($C_2$) and the upper right chain ($C_3$) are *y*-monotone since they are part of the right monotone polygonal chain. $\square$

##### *Remark*

It has been proved that the ordered points in the chains derived from a convex polygon, i.e., $C_1$, $C_2$, $C_3$, and $C_4$, are monotonic to the *x*-axis and *y*-axis; see Lemma 2. In other words, the *x*- or *y*-coordinates of the points in a single polygonal chain always increase or decrease. This property has been listed in Table 1. In SPA, the detecting and discarding of interior points are performed according to the above property.

**Fig. 7:**
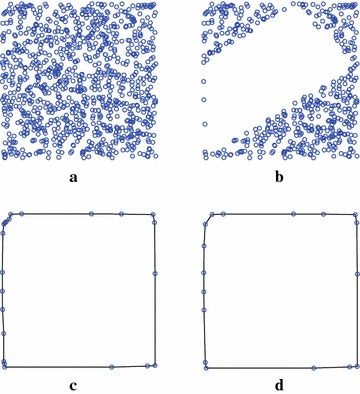
A simple example of computing the convex hull of 1000 points distributed in a *square*. **a** Input point set. **b** Remaining points after first filtering. **c** Remaining points after second filtering. **d** Convex hull

**Fig. 8:**
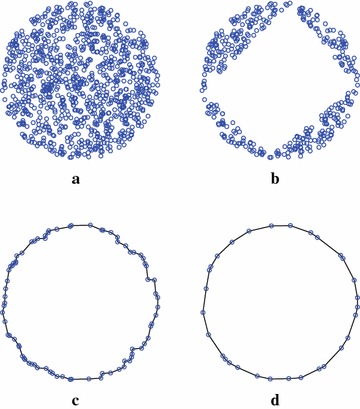
A simple example of computing the convex hull of 1000 points distributed in a *circle*. **a** Input point set. **b** Remaining points after first filtering. **c** Remaining points after second filtering. **d** Convex hull

However, a polygonal chain consisting of an ordered list of points that are monotonic to the *x*-axis and *y*-axis *cannot* be guaranteed to form a convex polygon or even a convex hull. Furthermore, even if a polygon is both *x*- and *y*- monotone, it cannot be guaranteed to be a convex polygon; see counterexamples in Figs. 7c and 8c.

The above behavior also suggests that: *NOT* all interior points can be identified and then discarded using the SPA; some interior points that are capable of satisfying the property of being both *x*- and *y*- monotone still exist.

In summary, (1) in each sub region, those remaining points that do not satisfy the property of being both *x*- and *y*- monotone MUST be interior points, and thus can be discarded; (2) in each sub region, those remaining points that satisfy the property of being both *x*- and *y*- monotone CAN be extreme or interior points.

#### The finalization of computing convex hull

After performing the SPA, typically four chains can be easily formed by simply connected the sorted points in each sub region, and then are used to form a simple polygon. The forming of the polygonal chains and simple polygon can obviously be achieved successfully. After that, the use of Melkman’s algorithm (Melkman 1987) can guarantee the success of finalizing the calculation of convex hull.

### Implementation details

In this section, more details about the implementation of the proposed algorithm will be described. The implementation has both the CPU side (host) and the GPU side (device) code. The code on the CPU side is developed to compute the convex hull of a simple polygon, which is relatively simple and easy to implement when compared to the code on the GPU side. Thus, implementation details are focused on the development of the GPU side code.

The implementation on the GPU side is developed by heavily taking advantage of the library *Thrust* for better efficiency and simplicity when using the data-parallel algorithm primitives. Currently, several GPU-accelerated libraries have been designed to provide data-parallel algorithm primitives such as parallel scan, parallel sort and parallel reduction. Such libraries include Thrust (Graham 1972; CUDPP 2015; CUB 2015). For that the library Thrust has been integrated in CUDA since version 4.0, it is very easy and convenient to use Thrust directly in CUDA. Hence, the library Thrust rather than the other two libraries are chosen to implement the proposed algorithms.

#### Performing the first round of discarding on the GPU

**Fig. 9:**
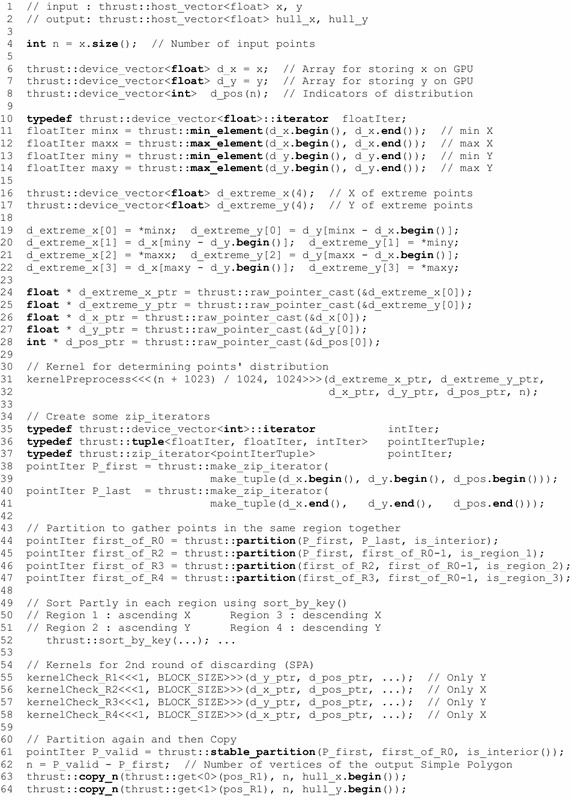
The implementation of the proposed algorithm (CudaChain)

The first step of discarding the interior points locating inside the quadrilateral formed by four extreme points is to find those points with the min or max *x*/*y* coordinates. In sequential programming pattern, a loop over all input points needs to be carried out to find the min or max values. In parallel programming pattern, the finding of min or max values in a vector can be efficiently achieved by performing a parallel reduction. Thrust provides such common data-parallel primitive and several easy-to-use interface functions. two functions, i.e., thrust::min_element() and thrust::max_element(), are used to efficiently find the min and max coordinates of all points in parallel; see lines 11–14 in Fig. 9.

To avoid the transformation between device memory and host memory, the memory addresses of the coordinates of extreme points and all input points that reside on the GPU are directly obtained using the function thrust::raw_pointer_cast(), and then pass as the launch arguments for the kernel kernelPreprocess; see lines 24–28 in Fig. 9.

**Fig. 10:**
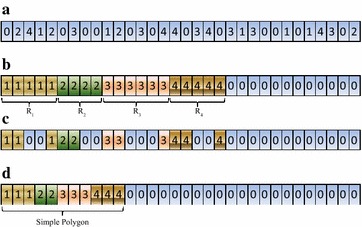
The second round of discarding interior points

A CUDA kernel, kernelPreprocess, is specifically designed to determine in which region a point falls. Each thread is responsible for calculating the position of a point; and the results are stored in an array d_pos[n]. The method for determining the distribution of points is introduced in “Algorithm design” section. An integer is used as an indicator of the position. For example, if a point $P_i$ locates inside the region $\hbox {R}_ 1$, then the value d_pos[i] is set to 1; and the indicator value of an interior point is 0. All the points that are not in the region $\hbox {R} _0$ are called exterior or remaining points. A simple example is presented in Fig. 10a.

#### Performing the second round of discarding on the GPU

The second round of discarding can be roughly divided into four steps: (1) perform four parallel partitioning for all points according to their positions, (2) sort the points in each region separately, (3) invoke a kernel for each region to discard the interior points using the method SPA, and (4) perform another parallel partitioning for all exterior points.

The first step, parallel partitioning, is carried out to gather those points in the same region together for subsequent procedure of sorting. After partitioning, the points that locate in the same region reside in a consecutive segment; see Fig. 10b. In this case, parallel sorting can be performed for each segment of points. Noticeably, it is decided to partition and sort each segment of points in place to minimize the cost of memory space.

After parallel sorting, a kernel is designed for each region to discard the interior points using the method SPA. There is only one thread block within the kernel’s thread grid. Each thread in the only thread block is responsible for checking consecutive (*m* + BLOCK_SIZE − 1)/BLOCK_SIZE points in the same region, where m is the number of points in a region for being checked, and BLOCK_SIZE represents the number of threads in the only block. In this implementation, BLOCK_SIZE is set to 1024 according to the compute capability of the adopted GPU. After checking and discarding interior points using SPA, some previous exterior points have been determined as interior ones; and their corresponding indicator values are modified to 0; see Fig. 10c.

In this implementation, only one thread block is allocated in the discarding of interior points using the proposed method SPA due to the data dependency issue in the discarding. When checking whether a point in a specific region such as the region $\hbox {R}_ 1$, the *y* coordinate of the point being checked is compared to that of the current last point of the formed chain; see Fig. 2a. This means the checking for a point, e.g., $P_i$, depends on the checking of the previous point $P_{i-1}$. It also means the checking for a set of consecutive points can only be performed in a sequential pattern. However, it is able to first divide a large set of consecutive points into some smaller subsets of consecutive points, and then perform the checking in parallel for each subset of points separately. This solution is in the divide-and-conquer fashion, which is also adopted in the proposed algorithm. However, it is not able to determine the optimal size of a subset of points or the number of all subsets. Thus, it is decided to divide a large set of consecutive points into BLOCK_SIZE subsets, while each subset contains (m + BLOCK_SIZE - 1)/BLOCK_SIZE points; and then, the checking for all the BLOCK_SIZE subsets is carried out in parallel.

The final step is to re-partition and copy the coordinates of the exterior points in current stage for outputting. Noticeably, to preserve the relative order of the sorted points, the function thrust::stable_partition() rather than thrust::partition() is used to compact the exterior points; see lines 60–64 in Fig. 9. After the stable partitioning, the remaining exterior points are stored consecutively and can be easily copied in parallel for being used on the host side (on the CPU); see Fig. 10d.

**Fig. 11:**
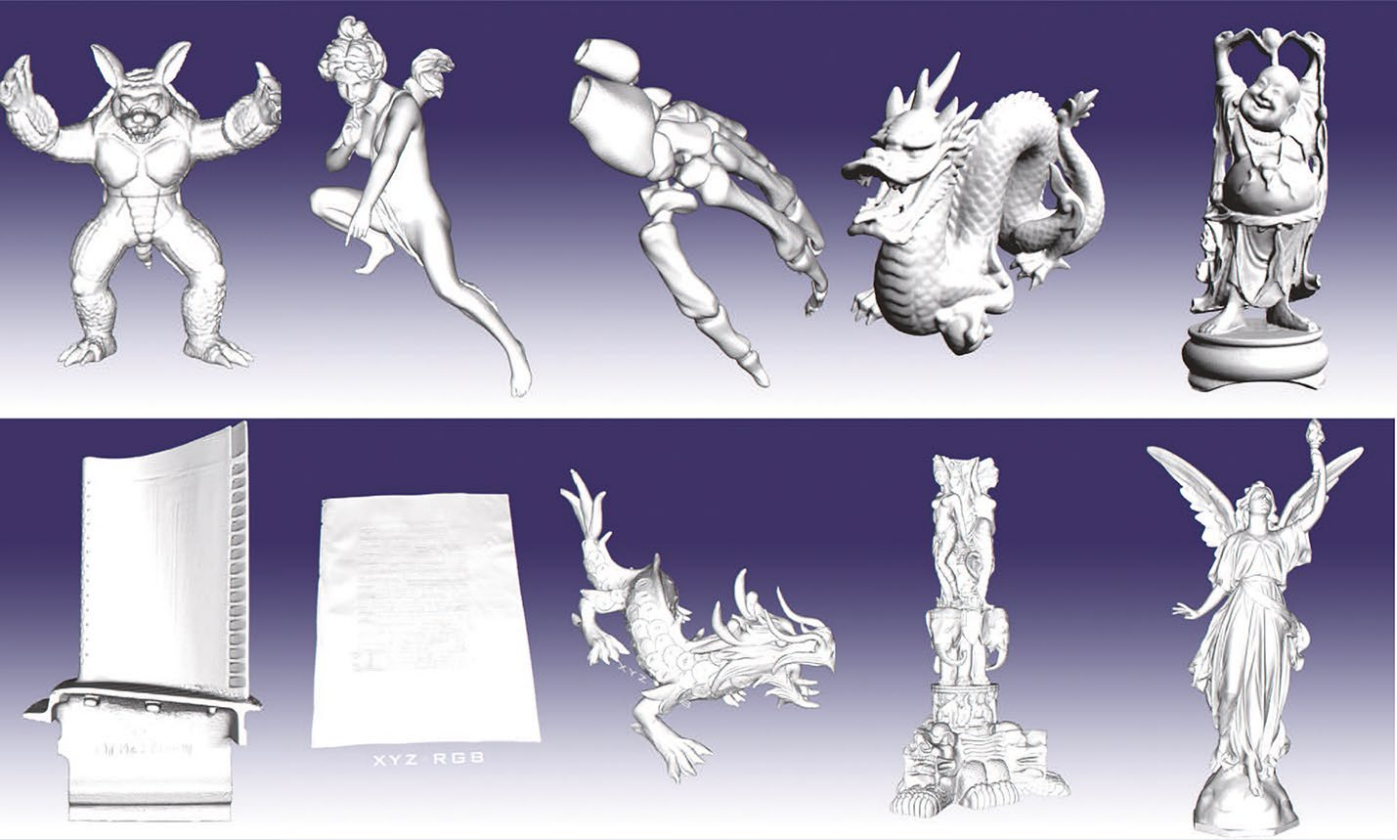
3D mesh models from Stanford 3D Scanning Repository and GIT large geometry models archive. From the *left to the right*, the models are: Armadillo, Angel, Skeleton Hand, Dragon, Happy Buddha, Turbine Blade, Vellum Manuscript, Asian Dragon, Thai Statue, and Lucy

**Fig. 12:**
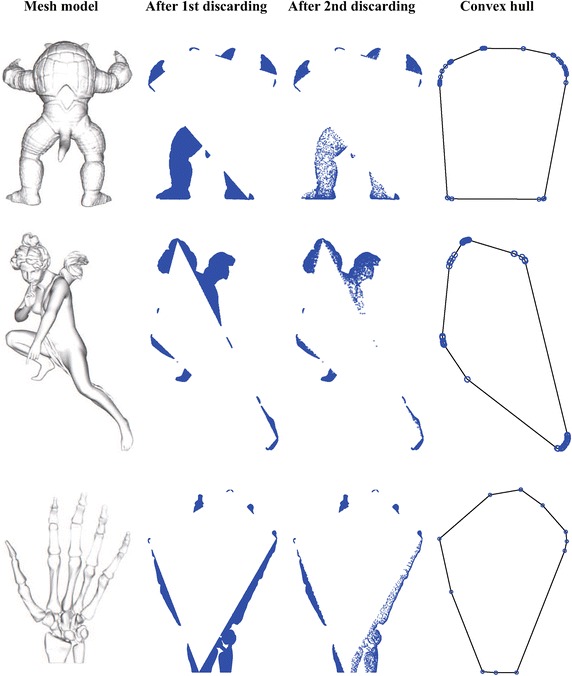
The calculating of convex hulls for the points derived from the models Armadillo, Angel, and Skeleton Hand

## Results

The proposed convex hull algorithm has been tested against the Qhull library (Qhull 2015) on various datasets of different sizes using two machines. The first machine features an Intel i7-3610QM processor (2.30 GHz), 6 GB of memory and a NVIDIA GeForce GTX660M graphics card. The other machine has an Intel i5-3470 processor (3.20 GHz), 8GB of memory and a NVIDIA GeForce GT640 (GDDR5) graphics card. The graphics card GTX 660M has 2 GB of RAM and 384 cores; and the GT640 graphics card has 1 GB of RAM and 384 cores. All the experimental tests have been evaluated using the CUDA toolkit version 5.5 on Window 7 Professional. Note that the complete source code, an input sample test data, and the corresponding output result are provided as the supplementary materials (see Additional files 1, 2, and 3).

Three groups of datasets have created for testing. The first group includes 8 sets of randomly distributed points in a square that are generated using the rbox component in Qhull. Similarly, the second group is composed of 8 sets of randomly distributed points in a circle. The third group consists of 10 point sets that are derived from 3D mesh models by projecting the vertices of each 3D model onto the XY plane. These mesh models presented in Fig. 11 are directly obtained from the Stanford 3D Scanning Repository (http://www.graphics.stanford.edu/data/3Dscanrep/) and the GIT Large Geometry Models Archive (http://www.cc.gatech.edu/projects/large_models/). Three application examples of computing the convex hulls for points derived from the mesh models Armadillo, Angel, and Skeleton Hand are presented in Fig. 12.

Note that the running time presented in this work includes the overhead of transferring data between the host side (CPU) and the device side (GPU).

**Fig. 13:**
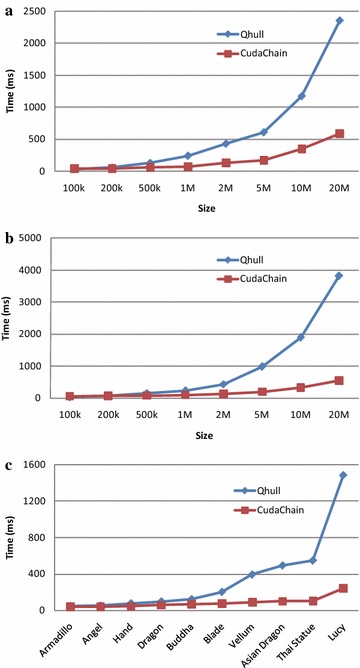
The efficiency of CudaChain on the GPU GTX 660M against CPU-based Qhull using the same datasets and the same machine. **a** Point sets distributed in *squares*; **b** Point sets distributed in *circles*; **c** Point sets derived from 3D models

**Fig. 14:**
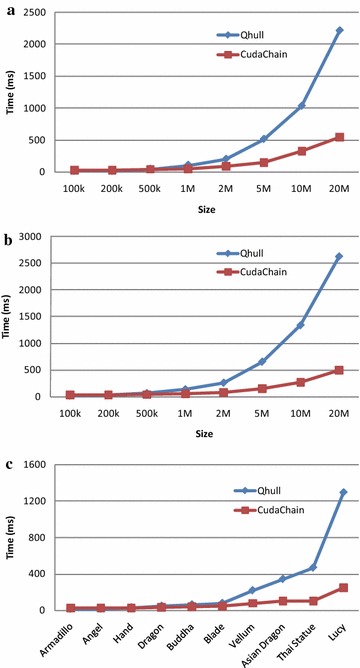
The efficiency of CudaChain on the GPU GT 640 against CPU-based Qhull using the same datasets and the same machine. **a** Point sets distributed in *squares*; **b** Point sets distributed in *circles*; **c** Point sets derived from 3D models

**Fig. 15:**
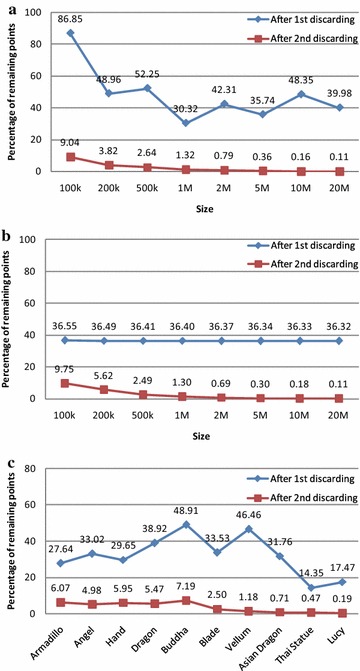
The effectiveness of two rounds of discarding interior points (*first round* of discarding: removing the points inside the quadrilateral formed by four extreme points. *Second round* of discarding: removing interior points according to the rules of the method SPA). **a** Point sets distributed in *squares*; **b** point sets distributed in *circles*; **c** Point sets derived from 3D models

**Table 1:** Regions and corresponding rules for discarding interior points

Region	First point	Last point	Order of *x* coordinates	Order of *y* coordinates	Rule for discarding
Lower left ($\hbox {R}_{1}$)	$P_{minx}$	$P_{miny}$	Ascending	Descending	Sort *x* in ascending order first; Discard point *P* according to the rule listed in Fig. 2a
Lower right ($\hbox {R}_{2}$)	$P_{miny}$	$P_{maxx}$	Ascending	Ascending	Sort *y* in ascending order first; Discard point *P* according to the rule listed in Fig. 2b
Upper right ($\hbox {R}_{3}$)	$P_{maxx}$	$P_{maxy}$	Descending	Ascending	Sort *x* in descending order; Discard point *P* according to the rule listed in Fig. 2c
Upper left ($\hbox {R}_{4}$)	$P_{maxy}$	$P_{minx}$	Descending	Descending	Sort *y* in descending order; Discard point *P* according to the rule listed in Fig. 2d

**Table 2:** Comparison of running time (/ms) for point sets distributed in squares on GTX 660M

Size	Qhull	CudaChain				Speedup
		Total	GPU	CPU	CPU(%)	
100k	27	42.5	39.6	2.9	6.82	0.64
200k	52	45.9	43.1	2.8	6.10	1.13
500k	124	65.6	61.4	4.2	6.40	1.89
1M	237	75.0	70.8	4.2	5.60	3.16
2M	426	129.1	123.2	5.9	4.57	3.30
5M	605	174.8	169.4	5.4	3.09	3.46
10M	1171	351.8	345.9	5.9	1.68	3.33
20M	2353	587.4	581.9	5.5	0.94	4.01

### Efficiency on the GTX 660M

The running time on the GPU GTX 660M of three groups of testing data, i.e., the group of randomly distributed point sets in squares, the groups of point sets in circles, and the group of point sets derived from 3D models, is listed in Tables 2, 3 and 4, respectively. To evaluate the computation load between the GPU side and the CPU side of the algorithm CudaChain, the running time is evaluated separately for both of the two sides and calculate the workload percentage of the CPU side; see Fig. 13.

For all the three groups of experimental tests, the experimental results show that: for small size of testing data, the Qhull is faster than the proposed CudaChain, while CudaChain is much faster than Qhull for the large size of testing data. The speedups of CudaChain over Qhull become larger with the increasing of the data size. The speedup is about 3×–4× on average and 5×–6× in the best cases.

The workload percentage of the CPU side is much smaller than that on the GPU side; and it decreases for the group of randomly point sets when the data size increases. In addition, the workload percentage of the CPU side is usually less than 10 %, except for the test of the model Happy Buddha.

Furthermore, the workload of three main steps, i.e., the first discarding, the second discarding, and the finalization of computing convex hull are evaluated for the third group of tests; see Table 5. The results indicate that: (1) the most computationally expensive step is the second one, while the computationally cheapest step is the third one; (2) when the size of input data becomes bigger, the workload percentage of the first step increases, and the workload percentage of the second step decreases.

**Table 3:** Comparison of running time (/ms) for point sets distributed in circles on GTX 660M

Size	Qhull	CudaChain				Speedup
		Total	GPU	CPU	CPU(%)	
100k	31	54.0	50.3	3.7	6.85	0.57
200k	62	65.5	61.2	4.3	6.56	0.95
500k	156	78.1	73.4	4.7	6.02	2.00
1M	225	95.0	90.2	4.8	5.05	2.37
2M	430	126.8	121.7	5.1	4.02	3.39
5M	982	193.0	187.5	5.5	2.85	5.09
10M	1897	317.9	311.5	6.4	2.01	5.97
20M	3811	543.6	536.6	7.0	1.29	7.01

**Table 4:** Comparison of running time (/ms) for point sets derived from 3D models on GTX 660M

3D Model	Size	Qhull	CudaChain				Speedup
			Total	GPU	CPU	CPU(%)	
Armadillo	172k	47	39.7	37.6	2.1	5.29	1.2
Angel	237k	51	41.6	38.7	2.9	6.97	1.2
Skeleton hand	327k	77	45.4	41.5	3.9	8.59	1.7
Dragon	437k	98	59.8	53.9	5.9	9.87	1.6
Happy Buddha	543k	123	68.7	59.6	9.1	13.25	1.8
Turbine blade	882k	202	73.9	67.5	6.4	8.66	2.7
Vellum manuscript	2M	392	90.6	86.9	3.7	4.08	4.3
Asian dragon	3M	492	101.7	97.9	3.8	3.74	4.8
Thai statue	5M	547	106.0	102.4	3.6	3.40	5.2
Lucy	14M	1481	245.2	240.9	4.3	1.75	6.0

**Table 5:** Workload of three main steps for point sets derived from 3D models on GTX 660M

3D Model	Size	Running time (/ms)				Percentage of running time		
		First step	Second step	Third step	Total	First step	Second step	Third step
Armadillo	172k	4.3	33.3	2.1	39.7	10.83	83.88	5.29
Angel	237k	4.8	33.9	2.9	41.6	11.54	81.49	6.97
Skeleton hand	327k	4.9	36.6	3.9	45.4	10.79	80.62	8.59
Dragon	437k	6.6	47.3	5.9	59.8	11.04	79.10	9.87
Happy Buddha	543k	7.3	52.3	9.1	68.7	10.63	76.13	13.25
Turbine blade	882k	8.4	59.1	6.4	73.9	11.37	79.97	8.66
Vellum manuscript	2M	11.7	75.2	3.7	90.6	12.91	83.00	4.08
Asian Dragon	3M	16.4	81.5	3.8	101.7	16.13	80.14	3.74
Thai statue	5M	21.0	81.4	3.6	106.0	19.81	76.79	3.40
Lucy	14M	60.7	180.2	4.3	245.2	24.76	73.49	1.75

(1) The first step is the first round of discarding on the GPU. The second step is the second round of discarding on the GPU. The third step is the finalization of computing the convex hull on the CPU. (2) The running time of the first step includes the overhead of transferring data from the host side to the device side; and the running time of the second step includes the overhead of transferring data from the device side back to the host side

### Efficiency on the GT 640

**Table 6:** Comparison of running time (/ms) for point sets distributed in squares on GT 640

Size	Qhull	CudaChain				Speedup
		Total	GPU	CPU	CPU(%)	
100k	15	25.5	24.2	1.3	5.10	0.59
200k	16	29.1	27.9	1.2	4.12	0.55
500k	47	40.4	38.4	2.0	4.95	1.16
1M	109	46.9	44.6	2.3	4.90	2.32
2M	202	83.5	81.2	2.3	2.75	2.42
5M	515	147.0	144.5	2.5	1.70	3.50
10M	1034	321.9	319.7	2.2	0.68	3.21
20M	2215	544.4	541.5	2.9	0.53	4.07

**Table 7:** Comparison of running time (/ms) for point sets distributed in circles on GT 640

Size	Qhull	CudaChain				Speedup
		Total	GPU	CPU	CPU(%)	
100k	16	25.9	24.4	1.5	5.8	0.62
200k	31	28.3	26.7	1.6	5.7	1.10
500k	62	33.3	31.7	1.6	4.8	1.86
1M	134	50.0	48.2	1.8	3.6	2.68
2M	258	74.6	72.6	2.0	2.7	3.46
5M	652	148.6	146.4	2.2	1.5	4.39
10M	1337	263.2	260.8	2.4	0.9	5.08
20M	2626	492.8	489.7	3.1	0.6	5.33

**Table 8:** Comparison of running time (/ms) for point sets derived from 3D models on GT 640

3D Model	Size	Qhull	CudaChain				Speed up
			Total	GPU	CPU	CPU (%)	
Armadillo	172k	15	26.5	25.0	1.5	5.66	0.6
Angel	237k	16	28.0	26.4	1.6	5.71	0.6
Skeleton hand	327k	31	29.6	27.8	1.8	6.08	1.0
Dragon	437k	47	35.1	31.8	3.3	9.40	1.3
Happy Buddha	543k	62	42.1	37.4	4.7	11.16	1.5
Turbine blade	882k	78	46.3	42.8	3.5	7.56	1.7
Vellum manuscript	2M	218	78.5	75.4	3.1	3.95	2.8
Asian Dragon	3M	343	102.5	98.8	3.7	3.61	3.3
Thai statue	5M	468	105.5	101.9	3.6	3.41	4.4
Lucy	14M	1295	248.8	244.5	4.3	1.73	5.2

On the machine with the GPU GT640, the running time of three groups of testing data is listed in Tables 6, 7 and 8. Similar to those experimental results obtained on the machine with the GTX 660M, for small size of testing data, the Qhull is also faster than the algorithm CudaChain, while CudaChain is much faster than Qhull for the large size of testing data. The speedups of CudaChain over Qhull also become larger with the increasing of the data sizes; see Fig. 14. The speedup is about 3×–4× on average and 4×–5× in the best cases. Noticeably, for the largest model Lucy, the speedup is 5.2× on the GT 640, while it is 6× on the GTX 660M.

The experimental results obtained on the machine with the GT 640 also indicate that: the workload percentage of the CPU side is much smaller than that of the GPU side; and it decreases for the group of randomly point sets when the data size increases. The behaviors are the same as those on the GTX 660M. Furthermore, the workload percentage of the CPU side is usually also less than 10 %, except for the test of the model Happy Buddha.

### Effectiveness of discarding interior points

 There are two rounds of discarding in the algorithm CudaChain. To evaluate the effectiveness of the proposed preprocessing method SPA, the remaining points after each round of discarding are counted; and then the effectiveness of two rounds of discarding is accordingly compared. The results presented in Fig. 15 show that SPA can dramatically reduce the number of remaining points and thus improve the overall efficiency of CudaChain. In addition, the effectiveness of discarding interior points by SPA becomes better with the increasing of the data size.

## Discussion

### Comparison

The algorithm CudaChain has been tested on two different machines with different GPUs. The efficiency performances of CudaChain on the two machines are almost the same. This result is due to the fact that the two GPUs, i.e., GTX 660M and GT 640, have the similar compute capability. However, the speedups of CudaChain over the implementation Qhull on two machines slightly differ. This behavior is lead by the different efficiency performance of CPU-based Qhull on the two machines.

One of the most important ideas behind our algorithm CudaChain is first to sort all points according to their *x* coordinates and then compute the convex hull of the sorted points in the Divide-and-Conquer fashion.

Both the sorting of points and the calculation of convex hull of sorted points are performed in parallel by exploiting the massively computing capability of modern GPU.

There exist some of other similar parallel algorithms designed for calculating the convex hulls of sorted points. In this subsection, we will theoretically analyze two classical ones of those parallel algorithms and compare with our algorithm.

Akl (1984) first presented an optimal parallel algorithm for sorting points in the plane, and then designed another optimal parallel algorithm for computing the convex hull of the sorted points in the plane. S. G. Akl also calculated the convex hull of sorted points in parallel by adopting the strategy of Divide-and-Conquer.

To calculate the upper hull or the lower hull, S. G. Akl first divides the input planar points into two subsets (i.e., the left and the right) with approximately equal size using a median vertical line, and then found the unique edge intersecting the vertical line, where the unique edge is formed by connected one point from the left subset and the other point from the right subset. The above procedure is recursively carried out until no more unique edges can be found.

Compare S. G. Akl’s algorithm with our algorithm, in both algorithms it needs to sort the planar input points in parallel according to the *x* coordinates. The essential difference between those two algorithms is the process of computing the convex hull of sorted points.

When computing the convex hull in S. G. Akl’s algorithm, the upper hull and the lower hull are separated calculated by finding the unique edges; and then the desired final convex hull is the merging of the upper hull and the lower hull.

In our algorithm, we also divide the potential convex hull into the upper and the lower ones; but further we split both the upper and the lower into the left part and the right part, and thus obtain four parts / chains, i.e., the right upper, the left upper, the right lower, and the left lower.

For each part, e.g., the right upper, we first filter / remove non-extreme points according to the sorted coordinates and then calculate the chain of the rest points. We do not directly find the chain of the upper hull or the lower hull, but first to remove some undesired points. In contrast, in S. G. Akl’s algorithm, the chain of the upper hull or the lower hull is directly found, i.e., the finding of unique edges.

Recently, Nakagawa et al. (2009) also developed a simple parallel algorithm for computing the upper hull of *n* sorted points. They first split the input sorted points into several subsets, then found the upper chain of each subset in parallel, and finally merged those upper chains using tangent edges to obtain the desired upper hull. They also employed a parallel algorithm to sort the planar input points before calculating the convex hulls.

Apparently, the strategy of Divide-and-Conquer is also utilized in Nakagawa’s algorithm.

Nakagawa’s parallel algorithm is computationally straightforward, and easy to implement on the multicore processors architecture. In addition, Nakagawa et al. (2009) demonstrated that their parallel algorithm can achieve acceptable speedups over the corresponding serial algorithm.

As mentioned above, in our algorithm, we do not directly obtain the upper hull or lower hull of the sorted points, but attempt to first remove some non-extreme points and then calculate the desired upper or lower hull / chain.

Similar idea is behind the Nakagawa’s parallel algorithm. They first divided the input sorted points into several subsets and then calculated the chain of each subset. In this procedure of calculating the chains, some non-extreme points are in fact checked and filtered. This is due to the fact that: if a point is not temporarily an extreme point of the “local” chain of the subset of points, then it is definitely not the extreme points of the “global” chain of all the points. Thus, in the parallel calculating of the “local” chains, some non-extreme points are implicitly determined and filtered.

In summary, all of our algorithm CudaChain, Akl’s algorithm (Akl 1984), and Nakagawa’s algorithm (Nakagawa et al. 2009) follow the Divide-and-Conquer paradigm. And these three algorithms are specifically designed to calculate the convex hulls of sorted points in parallel. The essential difference is the method of calculating the lower or the upper hull / chain.

Compared to other existing GPU-accelerated convex hull algorithms such as those implemented by parallelizing the QuickHull algorithm on the GPU (Srikanth et al. 2009; Srungarapu et al. 2011; Tzeng and Owens 2012), the algorithm CudaChain seems to be a bit slower than them. For example, Srikanth et al. (2009) reported that: compared the sequential QuickHull implementation, their implementation can obtain the speedups of about 10×–15×. It also has been introduced in Srungarapu et al. (2011) that: their implementation can achieve a speedup of up to 14× over a standard sequential CPU implementation. Tzeng and Owens (2012) declared that: they developed a parallel Quickhull implementation that can achieve an order of magnitude speedup over (Qhull 2015).

In summary, compared to those existing GPU-accelerated convex hull algorithms, CudaChain probably cannot achieve as high speedups as them, but is competitive in terms of the simplicity. More specifically, the main advantage of the algorithm CudaChain is that: it is very simple to implement and easy to use, which is mainly due to (1) the use of the library Thrust and (2) relatively less data dependencies. The data-parallel primitives such as parallel sorting and parallel reduction provided by Thrust are very efficient and easy to use; it is able to directly use these primitives in CUDA to realize the implementation without too many efforts. In addition, in CudaChain the only step that has data dependency is the checking and discarding interior points using SPA. Other steps or procedures can be very well mapped to the massively parallel nature of the modern GPU. This feature of having less data dependencies also makes CudaChain simple and easy to implement in practical applications. It hopes that the presented algorithm CudaChain is an alternative choice in practical applications for the trade-off between its simplicity and efficiency performance.

### Complexity and correctness

The time complexity in the worst case of the second round of discarding is *O*(*nlogn*) due to the sorting of points. Both the first round of discarding and the calculating of convex hull of a simple polygon run in *O*(*n*) . Thus, the worst case time complexity of the entire algorithm is *O*(*nlogn*) .

The space requirement of the algorithm CudaChain is also efficient. Only three arrays for storing all the input points’ coordinates and positions need to be allocated on the GPU. The parallel sorting, parallel reduction, and parallel partitioning completely operate on those three arrays in place without needing to explicitly allocate any additional global memory. In addition, to avoid the transformation from device memory and host memory and then back to device memory when invoking user-designed kernels, the memory addresses of those three arrays that resides on the GPU are directly obtained using the function thrust::raw_pointer_cast(), and then passed as the launch arguments for kernels.

The correctness of CudaChain can obviously be guaranteed. It is clear that: (1) in the calculating of convex hulls, any potential extreme points should not be discarded; and (2) any points that have been identified as interior ones can be discarded. As mentioned several times, there are two rounds of discarding in CudaChain. In the first round of discarding, those points locating in the quadrilateral formed by extreme points are definitely the interior ones and can be discarded. In the second round of discarding, It has been proved that the points detected as the interior using the proposed preprocessing method SPA is reasonable; see Fig. 3. Thus, this discarding can also be guaranteed to be correct. After two rounds of discarding, all the remaining points are used to calculate the expected convex hull.

In short, the proposed algorithm CudaChain can be guaranteed to be correct since (1) only those recognized interior points are removed and (2) all remaining points are preserved to avoid discarding any potential extreme points. More details on the proving of the correctness are presented in “Proof of correctness” section.

### Limitation

The first shortcoming of the algorithm CudaChain is that: the efficiency of discarding interior points using SPA within a single thread block cannot be guaranteed to be the highest. Probably, to hide memory latency and improve the efficiency, it needs to allocate several thread blocks in the discarding of interior points using the method SPA; and each thread is responsible for checking and discarding interior points for a small subset of consecutive points. However, the optimal number of consecutive points that assigned to be checked in each thread to generate the highest efficiency cannot be determined; this is due to the distribution of input points and the number of remaining points after the first round of discarding. It is probably to determine the optimal number of threads for a specifically distributed set of remaining points; but it is unable to do this for general cases. Thus, it needs more experimental tests to determine the optimal number of points that are assigned to each thread.

Another limitation is that: when all the input points are initially extreme points, e.g., when all points exactly locate on a circle, two rounds of discarding interior points will be wasteful since there are no interior points that can be found and removed. All the input points will be kept and used to calculate the convex hull on the CPU using the Melkman’s algorithm (Melkman 1987). Hence, the overall execution in this case might be very slow.

## Conclusion

In this paper, a novel sorting-based preprocessing approach (SPA) for discarding interior points and an alternative, GPU-accelerated algorithm, CudaChain, for calculating the convex hulls of planar point sets have been proposed. The correctness of the proposed algorithms have also been proved. The algorithm CudaChain is composed of two rounds of preprocessing procedures performed on the GPU and the finalization of calculating the expected convex hull on the CPU. The library Thrust has been utilized to realize the parallel sorting, reduction, and partitioning for better efficiency and simplicity. The proposed convex hull algorithm CudaChain has been tested against the Qhull library on various datasets of different sizes using two machines. Experimental results show that: (1) SPA can very effectively detect and discard the interior points; and (2) CudaChain achieves the speedups of 3×–4× on average and 5×–6× in the best cases. It hopes that the GPU-accelerated convex hull algorithm is an alternative choice in practical applications for the trade-off between its simplicity and efficiency.

When implementing the proposed algorithm, the library Thrust is heavily utilized. An efficient counterpart of Thrust, CUB (2015), has been developed recently. It was reported that CUB is faster than Thrust. It is expected to gain a significant increase in overall performance of the algorithm CudaChain by replacing Thrust with CUB. Future work should therefore include the implementation CudaChain using CUB and the evaluation of efficiency performance.

## Additional files

10.1186/s40064-016-2284-4 Source code of CudaChain.
10.1186/s40064-016-2284-4 A sample input test data.
10.1186/s40064-016-2284-4 The sample output result.
